# Anti-mullerian and androgens hormones in women with polycystic ovary syndrome undergoing IVF/ICSI

**Published:** 2013-11

**Authors:** Menha Swellam, Abeer Khaial, Tamer Mosa, Hatim El-Baz, Mohamed Said

**Affiliations:** 1 *Department of Biochemistry, Genetic Engineering and Biotechnology Research Division, National Research Center, Dokki, Giza, Egypt.*; 2 *Department of Biochemistry, El-Gouf University, Kingdom Saudi Arabia.*; 3 *Department of Clinical Biochemistry, Faculty of Medicine, North Jeddah Branch, King Abdulaziz University, Kingdom Saudi Arabia.*; 4 *Department of Obstetrics and Gynecology, Ain-Shams University, Cairo, Egypt.*

**Keywords:** *Polycystic ovary syndrome*, *Androgen hormone*, *Anti-mullerian hormone*, *Androstenedione*

## Abstract

**Background:** Despite its frequency, the polycystic ovary syndrome (PCOS) is still a difficult diagnosis in endocrinology, gynecology, and reproductive medicine. The Rotterdam consensus conference proposed to include the ultrasonographic follicle count as a new diagnostic criterion. Unfortunately, its assessment does not offer sufficient reliability worldwide**.**

**Objective: **To explore the possible roles of altered circulating androgens and anti-mullerian hormone among PCOS women regarding their body mass index and their outcomes after IVF.

**Materials and Methods:** In this cross sectional study, 195 women with PCO were included, they were divided according to their body mass index (BMI <27 kg/m^2^) as obese PCOS (n=91) and overweight PCOS (BMI ≥27 kg/m^2^) (n=104). Serum levels of androgens (dehydroepiandrosterone sulfate [DHEAS], testosterone and androstenedione [A4]), and anti-mullerian hormone (AMH) were assessed and compared with the endocrine profile and cycles outcomes.

**Results: **AMH, A4, FSH, and TSH concentrations were significantly higher in obese than in overweight women (p˂0.001). Contrary, LH: FSH ratio values, E_2_, PRL and DAHE-S levels were significantly lower in obese than in overweight women (p˂0.0001). Total oocyte retrieved, mature and fertilized oocyte were significantly higher in obese than in overweight women. Among pregnant obese PCOS women both AMH and A4 were significantly increased and DAHE-S was significantly decreased compared to pregnant overweight PCOS women.

**Conclusion:** Obese PCOS women have a higher chance of getting pregnant over those categorized as overweight PCOS. Also, androgens and AMH levels recommended to be considered in IVF attributes among obese and overweight PCOS women.

## Introduction

Polycystic ovary syndrome (PCOS) is one of the outstanding matters of endocrinological and gynecological investigation due to its complex pathogenesis and its multiple clinical expressions. One of the most stable findings of clinical research on PCOS is the higher luteinizing hormone (LH) levels and the higher values of the LH to follicle stimulating hormone (FSH) ratio. LH levels were found to be influenced by weight and, specifically, it has been repeatedly reported that normal-weight women with PCOS present significantly higher concentrations of LH compared to overweight and obese women with PCOS (1). 

Androgen-related disorders, such as polycystic ovary syndrome (PCOS), are the most common endocrine disorder in reproductive age women and affect approximately 7-12% of this population worldwide (1). Most recent research supports the opinion that androgen excess is a prerequisite diagnostic criterion for PCOS (2). Measurements of total testosterone (TT), androstenedione (A4) and dehydroepiandrosterone sulfate (DHEAS) are commonly used in research and clinical practice to identify women with hyperandrogenemia (2). 

Anti-mullerian hormone (AMH), also known as Mullerian-inhibiting substance, is a member of the transforming growth factor beta super family and is expressed only post-natally in the ovary and in granulosa cells of growing follicles (3, 4). Circulating concentrations of AMH are significantly higher in women with PCOS than in age-matched controls (5). 

It has been suggested that serum AMH concentrations correlate with the number of small pre-antral and early antral follicles. Serum AMH levels were also reported to correlate with elevated LH levels in PCOS, increased follicle number and ovarian volume on ultrasound examination (6). Though there have been several investigations into circulating AMH, and androgen hormones (TT, A4, and DHEAS) levels in patients with PCOS, no previous studies compared their concentrations in women with PCOS undergoing IVF/ICSI. 

The purpose of the current study was to assess the effect of the above mentioned circulating hormones and obesity among PCOS patients especially those undergoing IVF/ICSI and to compare these results with profile of endocrine hormones (LH, FSH, their ratios, PRL, E_2_, and TSH). 

## Materials and methods

**Study population selection and stratification**

Medical records were examined for patients who sought fertility consultation or treatment at the Infertility Division of the Department of Obstetrics-Gynecology, Ain Shams University Hospital during the period from December 2011 till November 2012. The study protocol was approved by the Medical Ethical Committee of Ain Shams University Hospital, Egypt, and was initiated after achieving written consent from the participants (n=195). 

The inclusion criteria were patients who fitted the medical definition of infertility “One year of unprotected intercourse but not pregnant”, with no previous in vitro fertilization (IVF) or intra-cytoplasmic sperm injection (ICSI) cycles, partner with normal semen parameters, while the exclusion criteria was the presence of 1) history of ovarian or adnexal surgery, 2) suspicious findings of ovarian malignancy, and 3) presence of endocrine disorders such as diabetes mellitus, hyper-prolactinemia, thyroid dysfunction, congenital adrenal hyperplasia, Cushing’s syndrome, and adrenal insufficiency. Diagnosis of PCOS was based on the revised Rotterdam criteria, as follows when two of the three following criteria are present: oligo/anovulation, clinical and/or biochemical signs of hyperandrogenemia, and polycystic ovaries (≥12 follicles measuring 2-9 mm in each ovary) (7, 8). 

None of the participants had galactorrhea or any systemic disease that could possibly affect their reproductive physiology. No woman reported use of any lipid-modulating medication or other substance that could interfere with the normal function of the hypothalamic-pituitary-gonadal axis. All patients consented after being fully informed. Additionally, all women were divided into two groups according to their BMI, namely obese (BMI ˂27 Kg/m^2^) (group A: n=91) and overweight (BMI ≥27 Kg/m^2^) (group B: n=104). 

**Ovarian stimulation protocols**

All women underwent controlled ovarian hyper-stimulation (COH) with gonadotropin (GnRH) long protocol approved by Assisted Reproductive Treatment (ART) Unit, Ain Shams University Maternity Hospital. All participants received folic acid 400 mg/day before initiation the induction cycle, combined oral contraceptive pills on day 3 of the previous cycle. Then standard mid luteal protocol starts with daily subcutaneous injection of triptoreline acetate, on the day 21 of the previous cycle. Estradiol (E_2_) was measured on the 2^nd^ day of menstrual cycle if it was less than 50 pg/ml, a daily human menopausal gonadotropin (HMG) (Ferring, Germany) or purified urinary FSH (Company, Country) injection was started. 

The starting dose of GnRH was prescribed according to age and body mass index (BMI) of the patients. PCOS patients have received 150 IU of human menopausal gonadotropin (HMG; Menopur; Ferring GmbH, Kiel, Germany) regardless of the age. Then at the 6^th^ day of stimulation the dose was adjusted according to ovarian response which was assessed by Transvaginal Ultrasound (TVU/S) (Siemens, Sonoline G20). After at least one follicle reached 14 mm in diameter, a daily injection of 0.25 mg of cetrorelix (Cetrotide; Serono, Baxter Oncology GmbH, Halle, Germany) was given until the day of HCG administration. When at least two follicles reached 18 mm in diameter, 10,000 IU HCG (Pregnyl; Schering-Plough, Kenilworth, NJ, USA) was administered and oocyte retrieval was performed 34-36 hours later. 

Conventional IVF or ICSI was conducted 4-6 hours post oocyte retrieval. For IVF, each oocyte was inseminated with 20×10^3^ motile spermatozoa in a single droplet containing 20 μl of fertilization medium (Quinn’s Advantage Fertilization medium; SAGE IVF Inc. Trumbull, Connecticut, USA). For ICSI, 1-2 μl washed spermatozoa were placed in 7% polyvinylpyrrolidone (PVP; SAGE IVF Inc.) and a sperm was injected into each denuded oocyte using standardized techniques. 

Each embryo was cultured in a single droplet containing 20 μl of medium (Quinn’s Advantage Cleavage medium; SAGE IVF Inc.) and incubated under the atmospheric composition of 5% CO_2_, 5% O_2_ and 90% N_2_ at 37^o^C. All embryo transfers were performed at 72 hours post oocyte retrieval.

**Luteal support and confirmation of pregnancy**

The luteal phase was supported by intramuscular injection of 50 mg of progesterone and vaginal supplementation of 300 mg micronized progesterone (Progeffik; Effik, Paris, France) or Crinone 8% progesterone gel (Columbia Laboratories, Inc., Livingston, NJ) once per day. 

Serum HCG was measured 14 days after oocyte retrieval and a value above 5 IU/ml was designated as positive pregnancy. Clinical pregnancy was defined as a pregnancy diagnosed by ultrasonographic visualization of the gestational sac. Viable pregnancy was defined as gestation age greater than 7^th^ weeks with documented fetal cardiac activity by ultrasound.

**Samples Collection**

On the day of oocyte retrieval and after overnight fast, the women underwent blood sampling by venipuncture at approximately 9:00 AM. Serum was separated and frozen in aliquots at -80^o^C for subsequent centralized analysis.

**Hormone examination**

Serum AMH levels were determined using a commercially available “second generation” enzyme-linked immunosorbent assay kit (Glory Science Co., Ltd, USA). Intra-assay and inter-assay coefficients of variation were <6% and <10%, respectively, with the lower detection limit at 0.13 ng/mL and linearity up to 21 ng/ml for AMH. Serum levels TT, A4, follicle stimulating hormone (FSH), luteinizing hormone (LH), thyroid-stimulating hormone (TSH), prolactin (PRL), and estradiol (E_2_) levels were determined with an automated multi-analysis system using a chemiluminescence technique (Advia-Centaur; Bayer Diagnostics, Puteaux, France). DHEAS (DRG Instruments GmbH, Marburg, Germany) were measured by enzyme-linked immunosorbent assay (ELISA) following the manufacturers’ instructions. 

**Statistical analysis**

Analysis of variance (ANOVA) was used to examine the effect of investigated hormones among PCOS group and BMI, results were reported as mean±SD. Also univariate analyses were performed using a Chi-square test. Simple linear regression analysis was used to establish the relationships among the investigated hormones. The level of significance (p) was determined to be less than 0.05. All analyses were performed using Statistical Package for the Social Sciences software 12.0 (SPSS Inc., Chicago, IL). 

## Results

**General features of the enrolled individuals**

A total of 195 participants were included in this study. The mean±SD (range) of women's age was 28.2±3.7 (20-35 years), the mean BMI was 27.6±4.2 (20-42 Kg/m^2^) at baseline (on cycle day 3 before COH). Controlled ovarian hyper-stimulation (COH) lasted 12.08±1.4 days, 116 out of 195 women developed to be clinically pregnant as reported in table I. 

**Endocrine profile and androgens levels among the studied population**

When the women in the current study were classified according to their BMI into obese (BMI˂27 kg/m^2^) (group A: n=91) and overweight (BMI ≥27 kg/m^2^) (group B: n=104) (Table II), it was found that FSH, TSH, AMH and A4 concentrations were significantly higher in obese than in overweight women (p=0.006, p=0.036 and p˂0.0001, respectively). Contrary, LH: FSH ratio values, E_2_, PRL and DAHE-S levels were significantly lower in obese than in overweight women (p˂0.01). The correlation between AMH and investigated androgens among the studied PCOS patients (n=195) revealed indirect correlation between AMH and DAHES (R=-0.289, p˂0.0001) and direct significant correlation between TT and A4 (R=0.49, p˂0.0001). 

Concerning overweight PCOS women (n=104), direct significant correlation was reported between DAHES and both TT and A4 (R=0.21 at p=0.05 and R=0.338 at p˂0.0001, respectively) and inverse significant correlation with AMH (R=-0.246, p=0.12). Testosterone was significantly correlated with A4 (R=0.552 at p˂0.0001). For Obese women no significant correlation between AMH and androgens was reported, apart from a significant correlation which was detected between TT and A4 (R=0.532 at p˂0.0001).

**Outcome of IVF/ICSI** **among the PCOS women regarding their obesity**

Total oocyte retrieved, mature and fertilized oocyte were significantly higher in obese than in overweight women as reported in table III. Although no significant level was reached, out of good quality of the emberyo (n=34), twenty (58.8%) were obese PCOS compare to 14 (41.2%) overweight PCOS. Also clinically pregnant was significantly increased in obese PCOS as compared to overweight PCOS ones. 

**AMH and androgens levels as regard to** **outcome of IVF/ICSI**

When authors compared the correlation between AMH, TT, A4 and DAHE-S among the outcome of IVF/ICSI regarding all investigated PCOS women (n=195), AMH showed no significant results with the outcome of IVF/ICSI, while TT, A4 and DAHE-S showed significant correlation (p˂0.0001) with retrieved oocyte, fertilized and mature oocyst. Among the entire PCOS group, for over-weight PCOS women (n=104) significant correlation was reported between TT, A4 and DAHES and retrieved oocyte, fertilized and mature oocyst (p˂0.0001), while AMH showed only significant inverse correlation with fertilized oocyt (R=-0.469, p˂0.0001). 

When considering the obese PCOS, a significant correlation was reported between TT, A4 and DAHE-S and fertilized and mature oocyst (R=0.322, 0.517 and 0.355, respectively at p˂0.0001), for AMH, a significant correlation with mature oocyte (R=0.268, p=0.01) was detected. For obese PCOS women AMH revealed inverse significant correlation with clinical pregnancy (R=-0.516, p˂0.0001) while TT and DAHES showed direct correlation (R=0.215, p=0.014, R=0.24, p=0.022, respectively). For overweight PCOS women, both TT and A4 showed inverse significant correlation (R=-0.201, p=0.04, R=-0.324, p=0.001, respectively). 

Among the entire groups, the levels of AMH and androgens for both obese PCOS and over-weight PCOS stratified by outcome of IVF/ICSI are shown in table IV. As authors were interested about PCOS women developed pregnancy (n=116), AMH and A4 were significantly increased (p˂0.001) in pregnant obese PCOS women (3.6±0.3, 3.2±0.78, respectively) versus pregnant overweight PCOS women (3.4±0.3, 2.7±0.8, respectively), while DAHES was significantly decreased (p˂0.001) in pregnant obese PCOS women (3.7±1.7) versus pregnant overweight PCOS women (4.8±2).

**Table I:** Characteristics features of the enrolled individuals

**Variable**		**PCOS (n=195)**	
		**Mean ± SD**	**95% CI**
Age		28.2 ± 3.7	27.7-28.7
BMI		27.67 ± 4.2	27-28.7
FSH (IU/L)		6.2 ± 1.7	6-6.4
LH (IU/L)		6.2 ± 2	6-6.5
LH / FSH		1.09 ± 0.4	1-1.16
PRL (IU/L)		13 ± 5.3	12.3-13.8
E_2_ (IU/L)		42 ± 16	39
TSH (IU/L)		2.2 ± 0.7	2-2.3
AMH (ng/ml)		3.6 ± 0.6	3.5-3.7
TT (nmol/L)		0.19 ± 0.61	0.16-0.2
A4 (nmol/L)		3.4 ± 2.6	3.1-3.9
DAHE-S (µ mol/L)\		4.1 ± 2	3.8-4.4
Total oocyte retrieved		16.3 ± 6.4	15.3-17.2
Mature oocyte		13 ± 5	12.2-13.6
Fertilized oocyte		10 ± 4	9.4-10.6
Embryo number		5 ± 2	4.6-5.2
Clinical pregnancy			
	Non-pregnant	79	
	Pregnant	116	

**Table II:** Clinical and endocrine parameters among investigated groups

**Groups**	**Obese PCOS ** **(n=91)**	**Over-weight PCOS** **(n=104)**	**p-value**
**Characteristics**			
Age (years)	28.6 ± 4	27.8 ± 3.6	0.153
FSH (IU/L)	6.59 ± 1.6	5.89 ± 1.8	0.006
LH (IU/L)	6.1 ± 1	6.3 ± 2.6	0.512
LH / FSH	1 ± 0.3	1.2 ± 0.4	0.005
E_2_ (IU/L)	38.5 ± 15	45 ± 17	0.006
PRL (IU/L)	11.6 ± 5.5	14.4 ± 4.9	˂0.0001
TSH (IU/L)	2.3 ± 0.6	2.1 ± 0.9	0.036
Serum AMH (ng/ml)	3.9 ± 0.4	3.3 ± 0.6	˂0.0001
TT (nmol/L)	0.18 ± 0.2	0.19 ± 0.1	0.968
A4 (nmol/L)	4.2 ± 3.7	3 ± 0.7	˂0.0001
DAHE-S (µ mol/L)	3.4 ± 1.7	4.7 ± 2	˂0.0001

Statistical analysis using analysis of variance (ANOVA).

**Table III:** Characteristics of outcome of IVF/ICSI among the PCOS women regarding their obesity

**IVF/ICSI outcome**		**Obese PCOS (n=91)**	**Over-weight PCOS (n=104)**	**p-value**
Total oocyte retrieved		17.6 ± 6.5	15 ± 6	0.006 a
Mature oocyte		14 ± 5	12 ± 4.8	0.008 a
Fertilized oocyte		11.3 ± 4.3	8.9 ± 3	˂0.0001 a
Embryo number		5.2 ± 2.5	4.55 ± 1.6	0.015 a
Embryo quality (n)				0.055 b
	Good (n=34)	20	14	
	Moderate (n=91)	40	51	
	Bad (n=70)	31	39	
Clinical pregnancy				0.048 b
	Non-pregnant (n= 79)	28	49	
	Pregnant (n=116)	63	53	

Statistical analysis using

a analysis of variance (ANOVA),

b Chi-square test.

**Table IV:** AMH, testosterone, androstatien and DAHE-S as mean±SD, stratified by outcome of IVF/ICSI in PCOS women

		**Obese PCOS (n=91)**				**Over-weight PCOS (n=104)**			
**IVF/ICSI** ** outcome **		**AMH** **(ng/ml)**	**TT** **(nmol/L)**	**A4** **(nmol/L)**	**DAHE-S** **(µ mol/L)**	**AMH** **(ng/ml)**	**TT** **(nmol/L)**	**A4** **(nmol/L)**	**DAHE-S** **(µ mol/L)**
Embryo quality									
	Good	3.4 ± 0.3 a	0.3 ± 0.1 a	3.9 ± 1.6 a	3.5 ± 0.3 a	3.8 ± 0.01 a	0.3 ± 0.1 a	2.25 ± 0.78 a	2.7 ± 1 a
	Moderate	3.8 ± 0.4	0.17 ± 0.12	5.8 ± 4.8	4.2 ± 2	3 ± 0.5	0.18 ± 0.09	2.81 ± 0.82	5.2 ± 2
	Bad	4.1 ± 0.3	0.13 ± 0.04	2.2 ± 1	2.4 ± 1	3.6 ± 0.6	0.15 ± 0.07	3.25 ± 0.56	4.6 ± 1.7
Chemical pregnancy									
	Non-pregnant (n= 65)	4.2 ± 0.5 a	0.14 ± 0.05	3.7 ± 2.1	3.1 ± 1.8	3.2 ± 0.9	0.24 ± 0.1 a	3.1 ± 0.8 a	4.3 ± 1.7
	Pregnant (n=130)	3.7 ± 0.3	0.2 ± 0.02	4.4 ± 4	3.5 ± 1.7	3.5 ± 0.2	0.16 ± 0.09	2.7 ± 0.7	4.8 ± 2
Clinical pregnancy									
	Non-pregnant (n= 79)	4.1 ± 0.5 a	0.13 ± 0.05 a	3.7 ± 1.8	3 ± 1.6 a	3.3 ±0.9	0.23 ± 0.13 a	3.2 ± 0.78 a	4.5 ± 1.6
	Pregnant (n=116)	3.6 ± 0.3	0.22 ± 0.02	4.5 ± 4	3.7 ± 1.7	3.4 ± 0.3	0.17 ± 0.1	2.7 ± 0.8	4.8 ± 2

Statistical analysis using analysis of variance (ANOVA),

a significant at p˂0.01.

## Discussion

PCOS affects 5-10% of women of reproductive age, making it the most common endocrine disorder of women in this age group. It is often seen in general internal medicine practice (9). The exact pathophysiology of PCOS and its initiating event have yet to be elucidated. However, various biochemical abnormalities have been described, and associations and linkages of one to another have been established (10, 11). Many of these abnormalities reinforce each other in vicious circles. Among them is a hypothalamic-pituitary abnormality such as elevated LH and low-normal FSH (12). 

In PCOS, the normal pulsatile secretion of LH is increased by an increased frequency and amplitude of pulses, while that of FSH is unchanged or muted. When authors grouped the enrolled PCOS patients into obese and overweight patients, FSH levels were significantly decreased in overweight PCOS patients compared to obese ones while LH: FSH ratio reported significant increase in overweight PCOS versus obese ones (12). In the current study elevated prolactin levels were reported in overweight PCOS versus obese ones. 

Extreme elevations of prolactin may stimulate adrenal production of DHEA-S which may reflect our finding that all overweight PCOS patients with PCOS have an increased sensitivity to androgens; androstenedione (of which >90% is produced in the ovaries), DHEAS (mainly produced in the adrenal glands) and testosterone (produced from the ovaries and adrenal glands in equal amounts) (13). In addition it has been reported that among the ovarian abnormalities in PCOS women the excess production of androstenedione which is produced by the ovarian stromal and the cal cells in response to LH. It is normally converted to estradiol by an FSH-dependent aromatase. 

Excess androstenedione in the circulation is converted to estrone, which exerts a tonic effect on LH production while contributing to a relative suppression of FSH production. In the face of a high LH: FSH ratio especially those overweight PCOS ones, more androstenedione is synthesized but is not aromatized, thus perpetuating a vicious cycle driving LH production and some prolactin production. In the current study, androstenedione was positively correlated with testosterone and this may be due to the fact that the ovary converts some androstenedione to testosterone, and in PCOS this is amplified. While DHEAS has little androgenic activity, small amounts can be converted to androstenedione and subsequently to testosterone. DHEAS has been traditionally used as the marker for adrenal androgen excess, because this hormone is: 1) 97-99% of adrenocortical origin, 2) the most abundant steroid, 3) relatively stable throughout the day and the menstrual cycle because of its relatively long-half life, and 4) is easily measured (14).

Significant elevation of estradiol was detected in overweight women compared to obese ones indicating that obesity can also be considered as a condition of increased estrogen production (15). The most frequently utilized laboratory test to examine ovarian reserve (OR) is anti-mullerian hormone (AMH). In the current study, AMH level was significantly higher in obese PCOS women as compared to overweight PCOS women (p˂0.0001). 

Among the enrolled PCOS patients AMH was negatively associated with DAHES (R=-0.289, p˂0.0001) these findings agreed with previous study. An explanation would be that the modification of adrenal androgens actively disrupts the usual mechanisms regulating the latter stages of follicular growth and development in PCOS. This, in turn, suggests possible effective inter-organ relationships. However, why or how adrenal androgen suppression should disrupt relationships while maintaining follicular profiles is difficult to explain (16). 

All the 195 PCOS (91 obese, and 104 overweight) enrolled patients underwent in vitro fertilization (IVF), among them 116 women reported clinically pregnant (63 obese and 53 overweight) also significant increment in IVF outcomes (oocyte yield, and embryo number and quality) was reported in obese PCOS patients compared to overweight ones. By considering the in vitro fertilization outcomes especially pregnancy chances, authors have detected in obese PCOS women an inverse significant correlation with AMH level and direct significant correlation with testosterone and DAHES. 

For overweight PCOS patients, an inverse significant correlation with testosterone and androstenedione. To our knowledge, this is the first report on determining androges and AMH as well among PCOS patients undergoing IVF and concerns about the impact of BMI indices on hormonal levels. Our preliminary findings indicate that obese PCOS women have a higher chance of getting pregnant over those categorized as overweight PCOS. Moreover, it should be pointed out that biochemical abnormalities accompany the PCOS should be taken into consideration in IVF attributes and that larger cohort studies with longitudinal follow up are needed to determine the possible role of androgens and AMH in women with PCOS especially obese women.

## Funding

This research did not receive any specific grant from any funding agency in the public, commercial, or not-for-profit sector.

## Conflict of interest

The authors have nothing to disclose.
